# Association Between Fat Graft Retention and Blood Flow in Localized Scleroderma Patients: A Pilot Study

**DOI:** 10.3389/fmed.2022.945691

**Published:** 2022-06-23

**Authors:** Hayson Chenyu Wang, Yunzhu Li, Zhujun Li, Liquan Wang, Ziming Li, Xiao Long

**Affiliations:** ^1^Department of Plastic and Reconstructive Surgery, Shanghai Ninth People’s Hospital, Shanghai Jiao Tong University School of Medicine, Shanghai, China; ^2^Department of Plastic Surgery, Peking Union Medical College Hospital, Peking Union Medical College and Chinese Academy of Medical Sciences, Beijing, China

**Keywords:** localized scleroderma, autologous fat grafting, microcirculation, blood flow, fat retention

## Abstract

**Background:**

Microcirculation plays a vital role in scleroderma physiopathology and the mechanism of fat survival.

**Objective:**

This study aims to assess the blood perfusion change after fat grafting and evaluate the relationship between blood perfusion and fat graft retention in patients with localized scleroderma (LS).

**Methods:**

A pilot study was conducted in patients with LS receiving autologous fat grafting (AFG). Fat graft retention measured by magnetic resonance imaging (MRI) analysis and blood flow perfusion measured by laser speckle contrast imaging 6 months postoperatively were noted. PUMC Localized Scleroderma Facial Aesthetic Index was used to assess the improvement of facial aesthetic impairment.

**Results:**

The fat retention at the 6-month follow-up was 34.56 ± 11.89 percent. At the 6th month of follow-up, the relative blood perfusion at the lesion area was 115.08 ± 14.39 PU, significantly higher than 100.42 ± 10.62 PU at the pre-operation (*p* = 0.010). The blood perfusion at follow-up increased by an average of 1.15 ± 0.14 times before the operation. No association between the increase in the blood flow perfusions and fat graft retention was found (*r* = −0.082, *p* = 0.811).

**Conclusion:**

Local blood perfusion in the lesion area relatively increased after AFG, but no direct relationship was found between fat retention and increased blood supply.

## Introduction

Localized scleroderma (LS) is a rare autoimmune connective tissue disorder characterized by inflammation and fibrosis of the skin and subcutaneous adipose tissues in the affected area (1, 2). Presenting as skin fibrosis, facial atrophy, and hyperpigmentation, LS seriously impairs the facial aesthetics and life quality of patients (1, 3). With multiple advantages, such as minor invasion and natural appearance outcomes, autologous fat grafting (AFG) has become the primary surgical treatment for patients with LS (1, 4, 5). AFG has been proved to produce aesthetic and therapeutic outcomes on patients with LS, including the clinical improvement of soft tissue atrophy, skin fibrosis, and pigmentation. However, the fat graft retention in patients with LS is often of a small amount due to the inflammatory microenvironment (5, 6). Microcirculation has been considered playing a vital role in the physiopathology of scleroderma and the mechanism of fat survival (7–9). However, no evidence has been obtained on blood flow and the effect of fat grafting in patients. This study aimed to assess the blood flow change after fat grafting and evaluate the relationship between blood flow and fat graft retention in the LS model.

## Patients and Methods

### Study Design and Intervention

A prospective pilot study approved by the institutional ethical committee was conducted in patients with LS seeking AFG to correct facial atrophy at Peking Union Medical College Hospital. All candidates were evaluated for suitability to be enrolled in the study by the criteria as follows. Inclusion criteria: (1) Patients with LS in the stable phase (documented by dermatologists); (2) Desire for correcting facial atrophy; (3) Signed informed consent. Exclusion criteria: (1) Absence of the lower abdomen fat donor site; (2) Undergoing steroid, anti-fibrotic or anti-scarring treatment (local or systemic); (3) Unstable or advanced phases of LS; (4) Chronic diseases that require to take long-term medication; (5) BMI > 2 points during the study.

The primary outcomes were the fat graft retention measured by magnetic resonance imaging (MRI) analysis and blood flow perfusion measured by laser speckle contrast imaging 6 months postoperatively. The secondary outcome was the improvement of facial aesthetic impairment of LS patients using PUMC Localized Scleroderma Facial Aesthetic Index.

### Liposuction, Graft Preparation, and Grafting

At study entry, the facial atrophy volume of the individual was measured by MRI analysis. By comparing the volume difference of the subcutaneous adipose tissue between the target lesion site and the unaffected symmetry site, the graft volume was noted for each participant (6). Liposuction at the abdomen was performed following Coleman’s method under a low, consistent negative pressure using a 3-mm cannula with 20-ml syringes to limit trauma to the adipocytes (10, 11). Before transferring, obtained adipose tissue was collected after sedimentation and filtration. Lysis of adhesion using a needle to penetrate multiple parallel tunnels under the skin was performed at local lesion before fat grafting. After the adhesion was removed, the fat graft was transferred into the subcutaneous tissue of the target lesion by a fan-like pattern technique using 1.4-mm blunt cannulas. A slight overcorrection of the facial atrophy was achieved (6, 11). The same surgeon performed all the grafting procedures in this study.

### Clinical Assessment

Preoperative variables included age, gender, duration, and BMI. Six months postoperatively, the participants were asked to return for follow-up assessments where surgical complications and BMI change were recorded, and the participants were photographed. Clinical assessment of facial deformity was separately evaluated by two plastic surgery experts using PUMC Localized Scleroderma Facial Aesthetic Index (PUMC LSFAI) preoperatively and postoperatively (3). PUMC LSFAI, with four domains for the local assessment and three domains for the overall assessment graded from 0 to 3, is a clinical evaluation tool designed to assess aesthetic impairment for patients with LS (3).

### Magnetic Resonance Imaging Analysis

**FIGURE 1:**
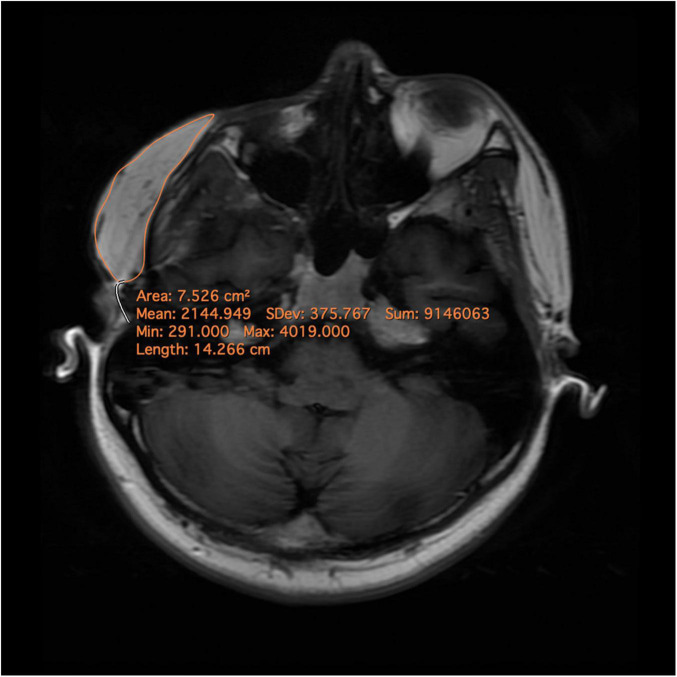
Magnetic resonance images and delineation of the subcutaneous adipose tissue. A region of interest (ROI) was precisely drawn on each axial slide by comprising pre- and post-operational scans. After ensuring that the entire targeted subcutaneous adipose tissue was included in the ROIs, the volumes were calculated automatically.

Magnetic Resonance Imaging (MRI) scan with a 3.0 Tesla MR scanner (Discovery MR750 3T, GE Healthcare, Milwaukee, WI, United States) was performed pre-operation, post-operation, and follow-up, using a thirty-two-channel head and neck coil. The MRI scan was conducted from the frontal bone to the neck base, excluding the supraclavicular region. Axial TSE T1-FLAIR weighted (acquisition parameters: TR = 1,725 ms, TE = 24 ms; flip angle, 111°; TA, 3 min 6 s; 50 slices; FOV, 22 × 20 mm) was used with a slice thickness of 4mm (6). Volumetric measurement was performed using Horos V3.3.1 (Horos Project, Annapolis, MD, United States) (Figure 1). The percentage change in the volume of adipose tissue of LS lesion was measured as follows:


$$\begin{array}{l} \text{Fat Retention =}\frac{\text{The volume of adipose tissue at follow up}-\text{The volume of adipose tissue preoperation}}{\text{The volume of adipose tissue postoperation}-\text{The volume of adipose tissue preoperation}}\times \text{100\%} \end{array}$$

The presence of complications was also assessed (6). All imaging was measured by an expert radiologist.

### Measurement of Facial Blood Flow Perfusion

**FIGURE 2:**
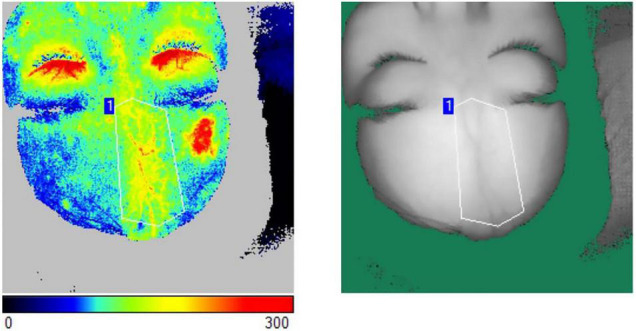
The measurement of blood flow perfusion was acquired by laser speckle contrast imaging. An ROI of the target area was precisely drawn. The absolute value of the blood flow perfusion at ROI was obtained by the acquisition.

Blood flow is a significant indicator to monitor the activity of LS and evaluate the efficacy of treatment (12, 13). Blood flow perfusion was measured on LS lesions pre-operation and follow-up by laser speckle contrast imaging (Pericam PSI System, Perimed, Sweden). The participant was instructed to lie down for a 10-min rest, after which the assessment began. The distance from the laser probe to the skin surface was set at 20 cm with a 785-nm laser wavelength, and the acquisition detection rate was three pictures per second (14). The measurement was carried out at the room temperature (15). After a region of interest (ROI) of the target area was precisely drawn, the absolute value of the blood flow perfusion at ROI was presented by a 20-s duration of acquisition (Figure 2). With a certain unaffected region fixed as a reference, the absolute values of the blood flow perfusion at the lesion area and the reference area were acquired (16). The relative blood flow perfusion at the lesion area was further obtained according to the following formula (16, 17):


$$\text{The relative blood flow perfusion at the lesion area =}\frac{\text{The absolute value of the blood flow perfusion at the lesion area}}{\text{The absolute value of the blood flow perfusion at the reference area}}\times \text{100 PU}$$

### Statistical Analysis

The sample size was estimated to be significant with 10 participants with an alpha of 0.05 and a beta of 0.8 by using PASS Statistical Software (NCSS, LLC). Statistical analyses were conducted using SPSS 26.0 (IBM, United States). Shapiro–Wilk test was used to assess continuous variables for normality. Paired *t*-test was used to assess normally distributed continuous variables, and results are presented as mean and standard deviation. Ordinal categorical variables were assessed using the Wilcoxon signed rank sum test, and results are presented as median and interquartile range. Spearman’s rank correlation coefficient was used to measure the association of non-normally distributed continuous variables or categorical variables. Significance was set to the level of *p* < 0.05.

## Results

Twenty-three patients (thirteen women and ten men) were enrolled in the study and retained throughout the 6 months to study completion, whose average age was 24.82 ± 5.93 years, and average BMI was 21.52 ± 1.77 kg/m^2^ (Table 1). Seventeen patients received AFG in the forehead, and six patients received AFG in the cheek. No patients were found with infection, hematoma, seroma, or other serious adverse events.

**TABLE 1:** Descriptive information of the participants.

Item	Number
Number	23
Male	10
Female	13
Age (yr)	24.82 ± 5.93
BMI (kg/m^2^)	21.52 ± 1.77
Duration (yr)	11.75 ± 3.55
**Lesion site**	
Forehead	17
Cheek	6
**Blood flow**	
Pre-operation (PU)	100.42 ± 10.62
Six-month follow-up (PU)	115.08 ± 14.39
Blood flow increase ratio	1.15 ± 0.14
Fat retention	34.56% ± 11.89%

*BMI, body mass index.*

The fat retention at the 6-month follow-up was 34.56 ± 11.89 percent. At the 6th month of follow-up, the blood perfusion at the lesion area was 115.08 ± 14.39 PU, significantly higher than 100.42 ± 10.62 PU at the pre-operation (*p* = 0.010). The blood perfusion at follow-up relatively increased by an average of 1.15 ± 0.14 times before the operation. Results from the univariable and multiple variable regression analyses on fat retention are presented in Table 2. Spearman’s correlation coefficient analysis further showed no association between the increase in the blood perfusion (the blood flow increase ratio) and fat graft retention (*r* = −0.082, *p* = 0.811). Clinical outcome assessment further showed the dyspigmentation, skin thickness, soft tissue atrophy, facial symmetry, facial proportion, facial profile, and PUMC LSFAI scores of the participants significantly improved at follow-up, except for the surface area of the lesion item (Supplementary Table 1). Spearman’s rank correlation coefficient analysis showed no association between fat retention and PUMC LSFAI scores (*r* = −0.014, *p* = 0.967).

**TABLE 2:** Regression analysis of fat graft retention at the follow-up.

Characteristic	Univariable analysis			Multiple variable analysis		
	Coefficient	95% CI	*p*	Coefficient	95% CI	*p*
Age	0.195	−0.009 to 0.016	0.566	0.787	−0.008 to 0.034	0.163
Gender	–0.027	−0.148 to 0.137	0.937	0.194	−0.190 to 0.264	0.677
Site	0.149	−0.126 to 0.189	0.663	–0.141	−0.243 to 0.183	0.718
Duration	0.414	−0.008 to 0.031	0.206	0.464	−0.012 to 0.038	0.231
BMI	–0.318	−0.058 to 0.022	0.341	–0.967	−0.121 to 0.013	0.088
Blood flow increase ratio	0.042	−0.492 to 0.551	0.902	0.797	−0.480 to 1.583	0.212

*BMI, body mass index.*

## Discussion

Localized scleroderma is a chronic benign inflammatory connective tissue disease that manifests as various plaques of different shapes and sizes with signs of skin inflammation, sclerosis, and atrophy (1, 2). A complex hypothetical mechanism of the pathogenesis of LS has been proposed, where vessels are affected and may, in return, contribute to its further development (5, 18, 19). The inflammatory reaction is the primary process in the early phase, attacking the vessels in the dermis and subcutaneous tissues and invading the adipose tissues (1, 5). The fibrosis process gradually takes place in the skin, with the progressive destruction of adipose tissues, resulting in the facial impairment of facial atrophy and depigmentation on the patient (2, 19).

Many studies have shown that local blood perfusion in the plaque is positively correlated with the activity of scleroderma; therefore, it can be used to predict disease progression and disease activity (7, 13, 20, 21). In the active inflammatory phase, the blood perfusion in the plaque increases, which is caused by inflammatory infiltration and vascular changes (13, 21). As the disease stabilizes, the blood flow tends to be normal, with no difference from the normal sites (14, 17).

In this study, we found that the blood perfusion in the lesion area relatively increased after AFG, which has never been reported in the literature. We reckon this result should not be interpreted as the reactivation of the disease as the patients’ symptoms did not worsen, and no occurrence of new lesions. The increase in local blood perfusion should be considered as a consequence of fat grafting, which is consistent with other studies (22). To date, two hypotheses of the mechanism of fat graft survival after transplantation have been proposed: graft survival theory and graft replacement (fat regeneration) theory (23). The mechanism of fat graft survival theory is based on the establishment of early blood circulation (23). Graft replacement theory believes that adipose-derived stem cells (ASCs) play a significant role in the graft replacement by promoting adipogenesis and angiogenesis (24). Both theories emphasize that the increase in local blood supply is beneficial and necessary to improve fat graft retention (9, 25, 26). Because adipocytes have poor tolerance to ischemic stress and undergo apoptosis and necrosis as early as 12 h under ischemia-mimicking conditions (27). ASCs can secrete multiple angiogenesis-related cytokines, including VEGF, HGF, GM-CSF, IGF-1, and PDGF, thereby prompting vascularization to increase fat survival and regeneration (24). Furthermore, we found that, although the local blood perfusion increased after AFG, the fat retention rate was not proportional to the increase in blood perfusion. This result indicates that fat retention involves many factors, and the increase in blood perfusion is only one part of it.

Another finding in this study was that AFG significantly improved facial aesthetics in many aspects, including soft tissue volume, skin thickness, pigmentation, facial symmetry, proportion, and profile. The increase in soft tissue volume directly results from AFG and further improves the overall facial aesthetics. In contrast, the skin texture and pigmentation changes may come from the regenerative effects of fat grafting (28). The regenerative effect of fat graft allows improvement of skin texture, including skin thickness and skin elasticity (29). Skin color modification can also be caused by diminishing local inflammation or by augmenting melanin (30). As proved in many disease models, such as aging and wound remodeling, these regenerative effects are considered the consequences of the paracrine function of ASCs (31).

Furthermore, previous studies on LS have shown ASCs had multiple advantages of immunomodulatory effects to limit inflammation and reduce fibrosis. By providing potent anti-inflammatory cytokines, such as interleukin-10, indoleamine 2,3-dioxygenase, and nitric oxide, ASCs have been proved to effectively disrupt the inflammatory response, inhibit the release of proinflammatory mediators, and decrease antigen presentation and phagocytosis, suppressing LS progression (32, 33). Meanwhile, increased anti-inflammatory cytokines also reduce the conversion of fibroblasts to myofibroblasts, thereby lessening the deposition of collagen in the skin and reversing the underlying fibrosis (34, 35). However, the area of lesions showed no significant improvement in this study due to the relatively insufficient follow-up time.

One of the limitations of this article is the small sample size. In addition, the measurement of the skin fibrosis and skin thickness of patients with LS was not achieved in this study. These indicators may represent the severity and prognosis of the disease, thereby facilitating the assessment of the outcome or reactivation of the disease after fat grafting.

## Conclusion

Autologous fat grafting significantly improved the impaired facial aesthetics, including soft tissue atrophy, skin thickness, dyspigmentation. Local blood perfusion in the lesion area relatively increased after AFG, but no direct relationship was found between fat retention and increased blood supply.

## Data Availability Statement

The raw data supporting the conclusions of this article will be made available by the authors, without undue reservation.

## Ethics Statement

The studies involving human participants were reviewed and approved by the Peking Union Medical College Hospital. The patients/participants provided their written informed consent to participate in this study.

## Author Contributions

HW and XL: conceptualization and supervision. HW: methodology, validation, and writing – original draft preparation. HW, YL, ZJL, LW, and ZML: formal analysis and investigation and resources. XL: writing – review and editing and funding acquisition. All authors contributed to the article and approved the submitted version.

## Conflict of Interest

The authors declare that the research was conducted in the absence of any commercial or financial relationships that could be construed as a potential conflict of interest.

## Publisher’s Note

All claims expressed in this article are solely those of the authors and do not necessarily represent those of their affiliated organizations, or those of the publisher, the editors and the reviewers. Any product that may be evaluated in this article, or claim that may be made by its manufacturer, is not guaranteed or endorsed by the publisher.
