# Supplementary material for: Association Between Fat Graft Retention and Blood Flow in Localized Scleroderma Patients: A Pilot Study
Source: Front Med (Lausanne). 2022 Jun 23;9:945691. doi: 10.3389/fmed.2022.945691 (PMC9259962; doi:10.3389/fmed.2022.945691)
Supplement: Supplementary file 1 [file Table_1.DOCX]

| Item | Pre-operation | Six-month follow-up | Z | P value |
| --- | --- | --- | --- | --- |
| Surface area of lesion | 1.5 (1.5-2.5) | 1.5 (1.0-2.5) | -0.378 | 0.705 |
| Dyspigmentation | 2.0 (2.0-3.0) | 1.5 (1.0-1.5) | -2.877 | 0.004 |
| Skin thickness | 2.0 (1.5-3.0) | 1.5 (1.0-2.0) | -2.565 | 0.01 |
| Soft tissue atrophy | 2.0 (1.5-3.0) | 1.0 (1.0-1.5) | -2.994 | 0.003 |
| Facial symmetry | 2.0 (1.5-2.5) | 1.5 (1.0-2.0) | -2.598 | 0.009 |
| Facial proportion | 1.5 (1.0-2.5) | 1.0 (0.5-1.0) | -2.401 | 0.016 |
| Facial profile | 1.5 (1.5-3.0) | 1.5 (1.0-2.0) | -2.388 | 0.017 |
| PUMC LoSFAI | 13.5 (12.5-16.5) | 9.5 (8.5-13.0) | -2.950 | 0.003 |

Supplement Table 1. The clinical assessment of facial deformity using PUMC Localized Scleroderma Facial Aesthetic Index.

Results are presented as median and interquartile range and assessed using the Wilcoxon signed rank sum test.
